# Vascular anomaly diagnosis by central venous catheter misplacement: a case report

**DOI:** 10.1186/s13256-022-03467-8

**Published:** 2022-06-22

**Authors:** Peter Paul de Smalen, Mark Jeroen Stoutjesdijk

**Affiliations:** 1 Department of Anesthesiology, Erasmus University Medical Centre, Na-17, Postbus 2040, 3000 CA Rotterdam, The Netherlands; 2 Department of Radiology, Ikazia Hospital Rotterdam, Rotterdam, The Netherlands

**Keywords:** Congenital heart disease, Cardiac vascular anatomy, Central venous catheter, Pulmonary hypertension

## Abstract

**Background:**

Congenital heart diseases rarely have a primary manifestation in adulthood. They are a rare cause of pulmonary hypertension in adults.

**Case presentation:**

A 70-year-old woman of Eurasian descent underwent emergency surgery for bowel ischemia. Her history of mild pulmonary hypertension likely correlates with a peculiar diagnosis of an anatomic anomaly on the postoperative x-ray and computed tomography scan. The central venous catheter was misplaced. Initial management consisted of removal of the catheter. The diagnosis, partial anomalous pulmonary venous return, may pose a clinical therapeutic dilemma.

**Conclusions:**

Partial anomalous pulmonary venous return is a potentially treatable cause of pulmonary hypertension. With the current trend toward more medical imaging, we expect this diagnosis to be made more often in the future.

## Introduction

Adult presentation of congenital heart disease is uncommon. Congenital heart diseases are a rare cause of pulmonary hypertension in adults. We describe a coincidental finding of partial anomalous pulmonary venous return (PAPVR) with pulmonary hypertension in the intensive care unit (ICU). Frequently, this condition is asymptomatic. However, with the current trend toward more medical imaging, we expect an increase in reported patients. This poses a therapeutic dilemma of whether and when to treat such patients.

Our patient gave written informed consent for publication. This manuscript adheres to the applicable EQUATOR guideline: ACRE checklist.

## Case presentation

A 70-year-old female of Eurasian descent presented to the emergency department with a left-sided stroke. She had medical history of obesity, hypertension, atrial fibrillation, insulin-dependent diabetes mellitus, chronic renal insufficiency, heart failure with preserved ejection fraction, and mild pulmonary hypertension. No interventions were possible for treatment of the stroke, and it was managed conservatively.

A week after admission, the patient developed increasing abdominal pain with melena. A computed tomography (CT) scan showed ischemia of the small intestines as a result of superior mesenteric vein thrombosis. A laparotomy was performed, and 60 cm of ischemic bowel was surgically removed. The attending anesthesiologist placed a central venous catheter (CVC) in the right internal jugular vein.

Postoperatively, the patient was admitted to the ICU. Vital signs recovered, and 2 days after surgery she was returned to the ward. Further diagnostics for coagulation disorders were initiated, after developing a stroke and mesenteric thrombosis under direct oral anticoagulation. Blood samples for Janus kinase (JAK2), calreticulin (CALR), myeloproliferative leukemia (MPL), homocysteine, and anti-cardiolipin antibodies were drawn. Anticoagulation therapy was changed to therapeutic doses of dalteparin and clopidogrel.

Six days after laparotomy, the patient developed increasing abdominal pain again. An emergency CT scan of the abdomen revealed occlusion of the distal superior mesenteric artery. Another laparotomy was performed, and a thrombectomy of the superior mesenteric artery was carried out, together with a revision of the anastomosis of the bowels. Unfortunately, the previously inserted CVC had already been removed. During surgery, a CVC was inserted into the left internal jugular vein and the patient was admitted to the ICU again.

**Fig. 1:**
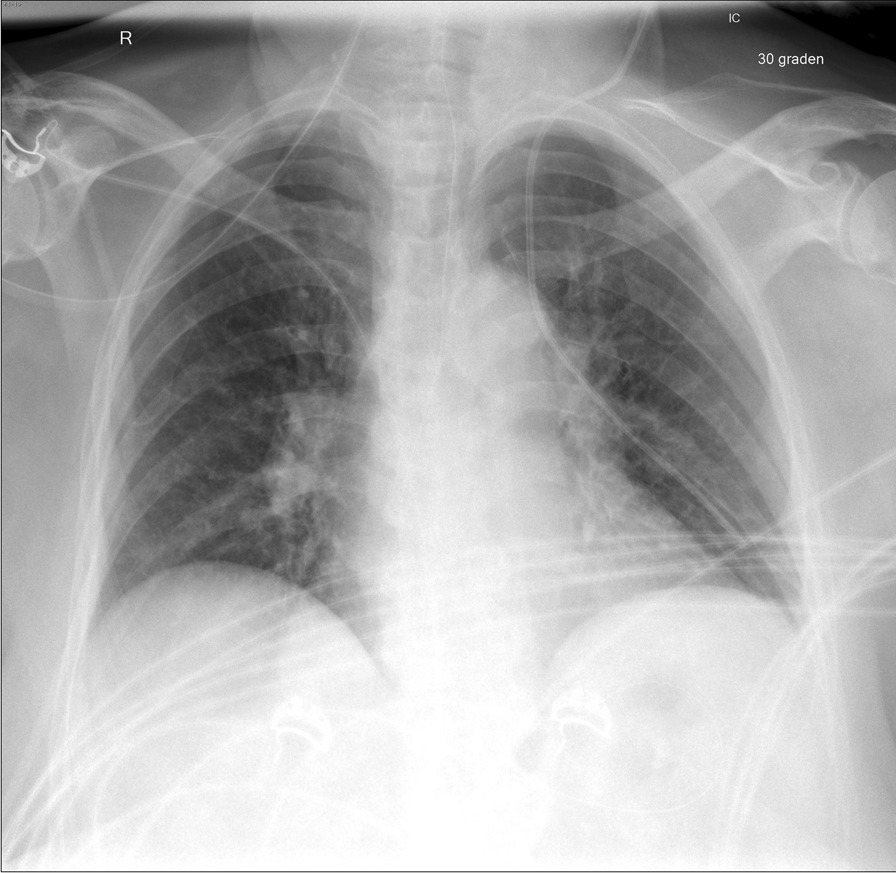
Chest X-ray showing the central venous catheter in abnormal position, peripherally in the left lung

**Fig. 2:**
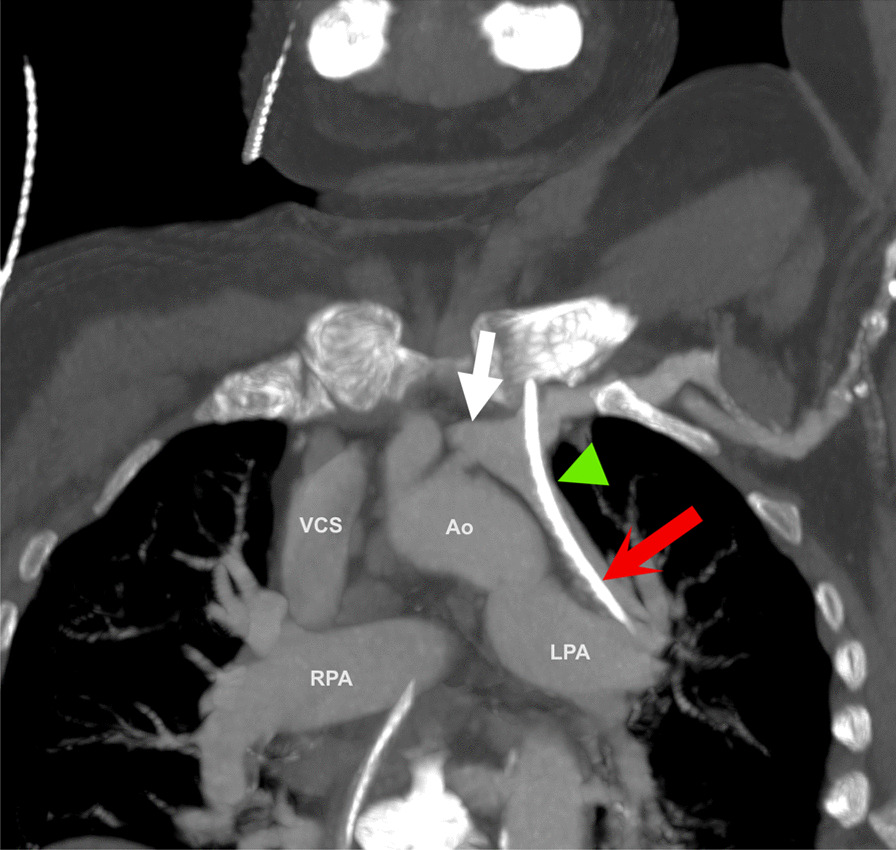
Multiplanar reconstruction image from the chest computed tomography examination. The intravenous catheter (red arrow) travels through the left jugular vein and would be expected to continue in the left brachiocephalic vein (white arrow). In this case, however, the route of least resistance was in the retrograde direction through the anomalous left superior pulmonary vein (green arrow head). Also visible are the superior vena cava (VCS), the aortic arch (Ao), and the left and right pulmonary artery (LPA, RPA)

Postoperative routine radiographic imaging for catheter placement showed the CVC in abnormal position (Fig. 1). A subsequent contrast-enhanced CT scan demonstrated a left-sided partial anomalous pulmonary venous return. The tip of the CVC was located peripherally in the venous drainage of the left lung. This vein was shown to drain into the left brachiocephalic vein (Fig. 2). The scan further showed minor parenchymal damage around the catheter tip. No associated other anomalies were identified. The CVC was removed, and a new one was placed in the right internal jugular vein. Three days after the second laparotomy, the patient was returned to the surgical ward. After her clinical condition improved, the patient was discharged to a rehabilitation center. Laboratory findings on coagulation disorders came back negative or normal.

Written informed consent forms were mailed to our patient, and her family helped complete the form.

## Discussion and conclusions

Complications after central venous catheterization are common. A 2003 review reports a risk of complications of more than 15% [1]. Pneumothorax, hematoma, and infection are the most common. It is estimated that CVC misplacement has a prevalence of 6.7% [2]. Cannulation of the left internal jugular vein and the left subclavian vein have the highest risk of misplacement. Guide wire complications are very uncommon but do occur [3]. Generally, the recommended management of catheter misplacement is removal and reinsertion at a different location.

PAPVR is a rare congenital heart disease with prevalence of approximately 0.2% on thoracic CT scans [4]. Normal venous drainage of the lung consists of four large veins that connect to the left atrium. Both the left and right pulmonary drainage consist of a superior and inferior vein that drain oxygenated blood towards the heart. Several variants of the normal anatomy are known [5].

Anomalous venous return of the right lung can drain into the superior or inferior vena cava, azygos vein, or coronary sinus. Anomalous venous return of the left lung can drain into the left brachiocephalic vein or coronary sinus. Most commonly, the right upper lobe drains into the superior vena cava (60–97%). This is associated with a sinus venosus atrial septal defect in 42% of cases [6]. A special case of right-sided PAPVR is Scimitar syndrome, in which a right-sided PAPVR is associated with hypoplasia of the right lung and pulmonary artery and dextroposition of the heart [7].

Turner syndrome is a genetic condition where one of the X chromosomes is (partially) missing. It occurs solely in females. Turner syndrome is associated with myriad congenital heart diseases. Among these heart diseases, PAPVR plays a dominant role. Eighteen percent of patients with Turner syndrome are diagnosed with PAPVR [8]. Other anomalies found are, for example, bicuspid aortic valve (12.5%) and aortic coarctation (6.9%).

Total anomalous pulmonary venous return (TAPVR) is a critical congenital heart defect that consists of an anomalous pulmonary venous return on both the left and right side. This condition is always symptomatic at birth.

Most patients with PAPVR are asymptomatic. The diagnosis is usually coincidental. Diagnosis of PAPVR can be difficult on transthoracic cardiac ultrasound. CT or magnetic resonance imaging (MRI) is more sensitive for its diagnosis. There has been a clear trend towards increasing use of CT and MRI scans in adults for a wide range of indications [9]. We therefore expect that the amount of asymptomatic patients diagnosed with PAPVR will continue to increase in a similar manner.

These incidental findings may pose a therapeutic dilemma. Anomalous venous return leads to a left–right shunt, and this produces a volume overload for the right ventricle. Therefore, PAPVR could play a significant role in the development of pulmonary arterial hypertension. This risk is increased as the amount of anomalous venous return increases. Surgical treatment of PAPVR could result in mitigation of the pulmonary hypertension. Thus, PAPVR is a rare cause of treatable pulmonary arterial hypertension [10]. However, surgical treatment carries significant risks of its own. Calculating the shunt fraction could aid in decision-making when surgical treatment is being considered [11].

This case of incidental PAPVR diagnosis after placement of a CVC is rare but not unique. In the past 20 years, a few such reports have been made [12, 13]. Management usually comprises removal of the catheter and placement of a new one. Clear recommendations regarding the follow-up and potential treatment of such patients are lacking.

Partial anomalous pulmonary venous return is a potentially treatable cause of pulmonary hypertension. Incidental diagnosis of this congenital anomaly at later age is expected to become more common as CT rates increase. This case reports describes the detection of an abnormal route of a central venous catheter as a more unusual way to arrive at this diagnosis.

## Data Availability

Not applicable.

## Abbreviations

Ao: Aorta
CALR: Calreticulin
CT: Computed tomography
CVC: Central venous catheter
ICU: Intensive care unit
JAK2: Janus kinase
LPA: Left pulmonary artery
MPL: Myeloproliferative leukemia
MPR: Multiplanar reconstruction
PAPVR: Partial anomalous pulmonary venous return
RPA: Right pulmonary artery
SVC: Superior vena cava
TAPVR: Total anomalous pulmonary venous return
